# Knowledge, attitudes and practices among Brazzaville midwives on cervical cancer screening

**DOI:** 10.11604/pamj.2020.36.311.19102

**Published:** 2020-08-20

**Authors:** Pierre Marie Tebeu, Jesse saint Saba Antaon, Derguedbé Nerbardoum, Pierre Vassilakos, Pierre de Beaudrap, Patrick Petignat

**Affiliations:** 1 Central Africa Inter-State Centre for Higher Education in Public Health, Cameroon,; 2 Faculty of Medicine and Biomedical Sciences, University of Yaoundé 1, Yaoundé, Cameroon,; 3 University Hospital of Geneva and Geneva Foundation for Medical Education and Research, Geneva, Switzerland,; 4 Research Institute for Development, Paris Descartes University, Paris, France,; 5 University of Geneva, Faculty of Medicine, Geneva, Switzerland

**Keywords:** Knowledge, attitude, practice, cervix cancer, midwives, Brazzaville

## Abstract

**Introduction:**

cervical cancer is an illness that causes 250,000 deaths worldwide. Data on Health professional's skills is highly important for the elaboration of prevention strategies. Objective: assess the knowledge, attitudes and practices (KAP) among Brazzaville midwives on cervical cancer screening.

**Methods:**

analytical KAP Study, from May 2^nd^ to August 10^th^ 2018. Participants are midwives working in the Gynaecology-Obstetrics departments of six hospitals in Brazzaville (Republic of Congo). Variables were related to their socio-demographic and occupational characteristics, as well as to their knowledge, attitudes and practices. Analyses were done using the Epi Info 7.2.2.6 software. Frequencies, central trend parameters, as well as rib ratios were calculated. Pearson, Fisher and Wald statistical tests with a significance level of 5% where used.

**Results:**

the study included 114 midwives aged 43.07 (± 7.40) years. They had an unsatisfactory level of knowledge (59.64%), favourable attitudes (92.98%) and poor practices (71.05%). The factors linked with best (satisfactory) knowledge were, seniority at workplace (10-27 years) [29.31%] vs. 51, 79%; OR; 2.59 (1.19-5.60)] and age (42-60 years) [31.81% vs. 52.08%; OR 2.32 (1.08-5.01)]. The best knowledge were related to the best practices (good) [16.18% vs. 47.83%; OR a = 2.95 (1.87-4.67)]; Midwives attitudes seem to not impact on their practices (p = 0.53).

**Conclusion:**

Brazzaville midwives have little knowledge and practices on cervical cancer screening. Therefore, the need of training them and equipping cervical cancer screening.

## Introduction

Precancerous cervical lesion is a benign and asymptomatic anomaly of the epithelium that may develop to invasive cervical cancer if no care [1]. Cervical cancer is the second leading cause of cancer deaths among women in resource-limited countries, just after breast cancer. It is a sexually transmissible disease, through human papillomavirus [1]. The development from precancerous status to invasive form takes 10 to 20 years [1]. The World Health Organization estimated that there were 500,000 new cases of cervical cancer and 250,000 subsequent deaths in the world [1]. Experts estimate that by 2030, deaths cause by cervical cancer will rise to more than 443,000 per year worldwide, and that 90% will be in African countries [2]. In 2015, GLOBACAN estimated they were more than 3,652 new cases of cervical cancer, with 2,862 deaths in Central Africa. During the same period the Republic of Congo registered 326 new patients and 165 deaths [1,3]. Few data on knowledge, attitudes and practices by Brazzaville midwives on cervical cancer are available. Objective: evaluate the knowledge, attitudes and practices of Brazzaville midwives on cervical cancer screening.

## Methods

This is an analytical KAP study, from May 2^nd^ to August 10^th^ 2018 in the six Brazzaville public hospitals (4 basic hospitals, UHC and Pierre Mobengo Hospital). The study included the midwives working in the Gynaecology-Obstetrics Services of the six Brazzaville public hospitals. Data were collected using a pretested survey questionnaire. It was organized in four sections, the first one focussing on socio-demographic and professional data. The second section focusing on knowledge of risk factors, on the extent of cervical cancer, on visual testing of precancerous lesions, on diagnostic tests, methods of treatment of precancerous lesions, on the means of treatment, the age group involved in the systematic of precancerous lesions screening. The third section focuses on midwives' attitudes towards cervical cancer screening; and the fourth section on their practice and attitudes towards cervical screening. Data on midwives' knowledge, attitudes and practice were marked with maximum scores 22 points, 4 points and 5 points, respectively. After marking each criterion, those of knowledge, as well as those of attitudes and practices were grouped together to obtain the number of points for each of three main variables (knowledge, attitudes and practices). The points scored made the categorisation of the knowledges, attitudes and practices into four levels. As for knowledge, they were considered very poor (0 - 5), insufficient (6 - 10), good (11 - 16), very good (17 - 22). Attitudes were categorized as follow: very negative (0 - 1), negative (1 - 2), positive (2 - 3), very positive (3 - 4). Practices were categorized as follow: very low (0 - 1), low (2 - 3), good (3 - 4) and very good (4 - 5). To assess the interaction between knowledge, attitudes and practices, the levels of knowledge, attitudes and practices were grouped in two ways each; for knowledge: unsatisfactory (0 - 10 points) and satisfactory (11 - 22 points); for attitudes: unfavourable ( 0 - 2 points) and favourable (3 - 4 points), and for practices: bad (0 - 3 points) and good (4 - 5 points). The data was analysed using Epi Info 7.2.2.6 software. As for the calculations carried out, in order to establish the levels of knowledge, attitudes and practices, absolute and relative frequencies as well as central trend parameters (mean, median) and dispersion (standard deviation and quartile) were calculated. As for the influence between the different variables, simple and multiple logistic regressions analysis was done and rib ratios were calculated with their confidence interval at 95%. Pearson, Fisher and Wald Statistical tests were used with a significance level of 5%.

## Results

The study included a total of 114 midwives, aged 28 - 60 years with an average of 43.07 (± 7.40) years and a median age of 41 (38 - 49). The midwives average seniority at the current workplace was 11.37 ± 7.76 years (Table 1). The analysis of knowledge gave the following categorisation among the 114 respondents in four levels: very insufficient (50.88 %); insufficient (8.77 %), good (38.60 %), and very good (1.75 %). Specifically, 22.81% of respondents acknowledge early sexual intercourse as a cervical cancer risk factor (Table 2, Table 3). By grouping knowledge into two levels (satisfactory & unsatisfactory). The factors connected with a high rating of having the best (satisfactory) knowledge were, seniority at work place (10 - 27 years) [29.31% vs 51, 79%; OR: 2.59 (1.19 - 5.60) and p = 0.01] and age (42-60 years) [31.81% vs. 52.08%; OR: 2.32 (1.08 - 5.01) and p = 0.029] (Table 4). As for midwives attitudes levels, the following was recorded: very negative (2.63 %); negative (4.39 %); positive (34.21 %), very positive (58.77 %) (Table 3, Table 4, Table 5). The analysis of midwifes practices reveals the following levels: very low (52.63 %); low (18.42 %); good (25.44 %), and very good (3.51 %) (Table 3). Specifically, 4.39 % of respondents reported having carried out a cervix screening (Table 6). Best practices (good) were connected to the best knowledge (satisfactory) and this finding remained unchanged after adjusting the confounding factors (age and seniority at workplace) on the level of knowledge. [16.18% vs. 47.83% ORa = 2.95 (1.87-4.67) and p = 0.001)]. Attitudes had no influence on practices (p = 0.53) (Table 7).

**Table 1:** socio-demographic and professional characteristics of Brazzaville midwives

Characteristics	Respondents N = 114	
	N	%
**Age**		
Average age (SD)	43.07 ± 7.40	
Median age (IQR)	41 (38; 49)	
Extreme age	(28, 60)	
**Age (group)**		
28 – 32	05	4.39
33 – 37	21	18.42
38 – 42	40	35.09
43 – 47	15	13.16
48 – 52	16	14.04
53 – 57	13	11.40
60	04	3.50
**Seniority at Current Work-Place (SCP) (years)**		
SCP average (SD)	11.37 ± 7.76	
SCP median (IQR))	10 (7;14)	
SCP extreme (years)	(2; 26)	
**(SCP) (group)**		
0 – 4	20	17.54
5 – 9	38	33.33
10 – 14	38	33.33
15 – 19	12	10.53
20 – 27	06	5.27
**Have participated in a training**		
Yes	04	3.51
No	110	96.49

**Table 2:** midwives knowledge on cervical cancer epidemiology, visual tests, and diagnostic

Midwives knowledge on cervical cancer	Respondents N = 114	
	n	%
**Cervical cancer: public health problem in the Republic of Congo (Brazzaville)**		
Yes	108	94.74
No	06	5.26
**Ranking of cervical cancer in the Republic of Congo**		
2^nd^	32	28.07
3^rd^	37	32.46
I don't know	45	39.47
**Cervical cancer risk factors**		
HPV infection	05	4.38
Too early sexual intercourse	26	22.81
Several sexual partners	39	34,21
High parity and too early sexual intercourse	02	1.75
Smoking	00	0.00
Age	03	2.63
Lack of screening	00	0.00
Immune deficiency	00	0.00
I don't know	31	27,20
Others 1	08	7.02
**Visual tests for precancerous lesions detection**		
VIA and VILI	17	14.91
I don't know	97	85.09
**Means of treatment for precancerous lesions**		
Cryotherapy	4	3. 51
I don't know	110	96.49
**Means of diagnosis for cervical cancer**		
Colposcopy	29	25.44
Uterine cervical smear	48	42,10
I don't know	37	32.46
**Means of treatment of cervical cancer**		
Surgery	36	31.58
Chemotherapy	30	26.32
Radiotherapy	18	15.79
Surgery and chemotherapy	11	9.65
Surgery and radiotherapy	08	7.01
Chemotherapy and radiotherapy	01	0.88
Surgery, chemotherapy and radiotherapy	01	0.88
I don't know	09	7.89
Others * 1: oral contraception		

**Table 3:** distribution of Brazzaville midwives surveyed by level of knowledges, attitudes and practices

Level of knowledge, attitude and practices	Score	Respondents N = 114	
	N	N	%
**Level of knowledge**			
Very insufficient	0 – 5	58	50.88
Insufficient	6 – 10	10	8.77
Good	11 – 16	44	38.60
Very good	17 – 22	02	1.75
**Level of attitude**			
Negative	0 -1	3	2.63
Few negative	2	5	4.39
Positive	3	38	34,21
Very positive	4	67	58.77
**Level of practice**			
Very weak	0 – 1	60	52.63
Low	1 – 2	21	18.42
Good	2 – 3	29	25.44
Very good	3 – 4	04	3.51

**Table 4:** socio-demographic, professional characteristics on respondents' level of knowledge

Characteristics	Total	Level of knowledge			
		Satisfactory	Unsatisfactory		
	N = 114	%	N=46	%	N=68	%	OR (95% CI)	P
**Age (group)**								
28 – 41	66	57.89	21	31.82	45	68.18	1	
42 – 60	48	42.11	25	52.08	23	47.92	2.32 (1.08 - 5.01)	0.02
**Seniority (group)**								
0 – 9	58	50.88	17	29.31	41	70.69	1	
10 – 27	56	49.12	29	51.79	27	48.21	2.59 (1.19 - 5.60)	0.01

**Table 5:** Brazzaville midwives attitudes on cervical cancer screening

Attitudes of midwives	Questioned person N = 114	
	n	%
**Seriousness of cervical cancer**		
Yes	112	98.25
No	02	1.75
**Importance of screening cervical cancer**		
Yes	112	98.25
No	02	1.75
**Purpose to talk of screening to women**		
Yes	73	64.04
No	41	35.96
**Role of midwives in reducing cervical cancer morbidity and mortality**		
Yes	106	92.98
No	08	7.02

**Table 6:** distribution of Brazzaville midwives practices on the prevention of cervical cancer

Midwives' practices	Respondents N = 114	
	N	%
**Have done cervical cancer screening**		
Yes	5	4.39
No	109	95.61
**Test already used**		
VIA or VILI	3	2.63
Uterine cervical smear	2	1.75
Never used	109	95.62
**Patient screening council**		
Yes	48	42.11
No	66	57.89
**Proposal cervical cancer screening**		
To women aged 25 – 65	10	8.77
To all women	25	21.92
To women bleeding	9	7.89
Others*	4	3.50
I don't know	66	57.89
Others* : women under 25 years old		

**Table 7:** Brazzaville midwives´ level of knowledge and attitudes´ impact on their of practices

Performance		Midwives practices				Crude		adjusted	
	Total	good		Bad		OR C CI 95%	P	OR has 95% CI	P
	N = 114	N = 33	%	N = 81	%				
**Knowledge**									
Satisfactory	46	22	47.83	24	52.17	4.75 (1.99 - 11.30)	0.01	2.95 to ( 1.87 - 4.67)	0.01
Unsatisfactory	68	11	16.18	57	83.82	1			
**Attitudes**									
Positive	106	9	8.49	97	91.51	1			
Adverse	8	1	12.50	7	87.50	1.53 (0.2 - 13.9)	0.53		

## Discussion

This study grants an overview on Brazzaville's midwife's skills on cervical cancer. However, it has some limitations, given that some midwives were unavailable when the investigation. The average age of midwives was 43.03 ± 7.40 years. Some investigations were carried out in the subject [4]. They recorded an average age lower than ours, i.e.; 36 years [4]. The findings lower than ours could be explained by the fact that, the participants of their studies included trainees and volunteers, who are generally younger than the permanent staff of our study. Analysing the knowledge; we found that: 22.81% of respondents reported early sexual intercourse as one of the cervical cancer risk factors; a low rate could be explained by the fact that the training programs for midwives do not include detailed modules on cervical cancer screening. Another possible explanation is that cervical cancer refresher courses are not carried out, in this background where there is no any national program against cervical cancer. A French study on midwifes knowledge and skills on cervical screening reported a higher rate than ours (27.4%). 27.90% of the French midwives, considered reported having received additional training in cervical cancer screening [5]. Only 3.50% of the contributors to our study reported having participated in a cervical cancer screening training. By categorising the respondent´s knowledge into four levels, the following was recorded: insufficient, 50.88% (58/114); average at 8.77% (10/114); good at 38.60% (44/114), very good 1.75% (2/114). We could not find studies on midwives' knowledge levels on cervical cancer screening. Our findings stress on the need to train midwives on cervical cancer screening.

The factors connected to best (satisfactory) knowledge were: seniority at the workplace (10 - 27 years), [OR: 2.59 (1.19-5 .60)]; age of midwives (42-60 years), [OR: 2.32 (1.08-5.01)]. These findings could be explained by the fact that a staff with several years of practice might have acquired good professional skills. Team working could also contribute to, as source of knowledge sharing. The analysis of the attitude shows that 98.25 % of respondents had a favourable opinion of cervical cancer screening. This finding could be explained by the fact that the midwives investigated generally considered cervical cancer a very dangerous disease. Other investigation were carried out on the same subject [6,7]. Some findings are similar to ours, i.e. 94.7% [6]. Some of them are lower than ours, 57.38% [7]. We found that 4.39% of midwives reported having carried out a cervical cancer screening. This finding could be explained by the fact that most of the hospitals in which respondents work do not have any screening units. Other studies were carried out on the same subject [6,7]. Their findings were higher than ours, ranging from 45.6% to 49.6%. These findings, which are higher than ours, could be explained by the fact that most Brazzaville basic hospitals do not have a cervical cancer screening unit, and almost all the respondents were not trained on cervical cancer screening, 95.61% (109/114).

By grouping the levels of practice in two categories, we found that 71.05% (81/114) had a poor level of practice on cervical cancer screening. We could not find studies on midwife's levels of practice for cervical cancer screening. The poor level of practices (71.05%) suggests the need to create screening units in Brazzaville hospitals, with trained midwives. This may help to limit suffering and deaths. Midwives with better knowledge had higher possibility of having best practice [OR a = 2.95 (1.87 to 4.67) and p = 0.001]. Therefore, good practices basement leads to good knowledge. This observation is consistent with the Health Belief Model Theory, according to which people´s practices depend on their knowledge. Henceforth, the obvious need to train midwives on cervical cancer screening. Other studies have also been carried out on the influence of knowledge on health professionals practices, related to breast cancer. They showed that good knowledge leads to good practice [8].

## Conclusion

Brazzaville midwives have little knowledge and practices on cervical cancer screening. Therefore, the need of training them and equipping cervical cancer screening.

### What is known about this topic

- The existence of cervical cancer is known;
- The consequences of cervical cancer are also known.

### What this study adds

- This study highlights the evaluation of knowledge, attitudes and practice of midwives on cervical cancer screening;
- It revealed that the acquisition of the best midwives on cervical cancer screening would allow good practice.
